# Optimizing the De-Noise Neural Network Model for GPS Time-Series Monitoring of Structures

**DOI:** 10.3390/s150924428

**Published:** 2015-09-22

**Authors:** Mosbeh R. Kaloop, Jong Wan Hu

**Affiliations:** 1 Department of Civil and Environmental Engineering, Incheon National University, 12-1 Songdo-dong, Yeonsu-gu, Incheon 406-840, Korea; 2 Department of Public Works and Civil Engineering, Faculty of Engineering, Mansoura University, Mansoura 35516, Egypt; E-Mail: mosbeh.kaloop@gmail.com; 3 Incheon Disaster Prevention Research Center, Incheon National University, 12-1 Songdo-dong, Yeonsu-gu, Incheon 406-840, Korea

**Keywords:** GPS, de-noise, monitoring, neural network

## Abstract

The Global Positioning System (GPS) is recently used widely in structures and other applications. Notwithstanding, the GPS accuracy still suffers from the errors afflicting the measurements, particularly the short-period displacement of structural components. Previously, the multi filter method is utilized to remove the displacement errors. This paper aims at using a novel application for the neural network prediction models to improve the GPS monitoring time series data. Four prediction models for the learning algorithms are applied and used with neural network solutions: back-propagation, Cascade-forward back-propagation, adaptive filter and extended Kalman filter, to estimate which model can be recommended. The noise simulation and bridge’s short-period GPS of the monitoring displacement component of one Hz sampling frequency are used to validate the four models and the previous method. The results show that the Adaptive neural networks filter is suggested for de-noising the observations, specifically for the GPS displacement components of structures. Also, this model is expected to have significant influence on the design of structures in the low frequency responses and measurements’ contents.

## 1. Introduction

The extracted displacement components of structures are one of the vital structural health monitoring (SHM) parameters, which can be used to predict the behavior of different types of civil structures, such as dams, bridges, and tall building. With large oscillations occurring during extreme natural hazards like earthquakes, landslides, wind-storm, surface subsidence and traffic accidents, etc., there is a high probability of potential disaster. Furthermore, the understanding of the response of structures to dynamic effects and, especially, the measurement of their displacements and dynamic parameters are of cardinal importance for structural engineering due to four reasons [1]: First, to design for dynamic characteristics, second, to understand the response of structures to dynamic loads, third, the monitoring of the structural health, and finally, new trends for displacement-based design of earthquake-resistant structures. The behavioral monitoring is mainly concerned with displacement and dynamics or oscillations of structure. Traditionally, the accelerometers are used to measure the dynamic parameters, and rarely depend on strain gauges, displacement transducers and more recently fiber optic sensors [1,2,3]. The displacement measurement is the prime problem for these methods. A real-time monitoring system using Global Positioning System (GPS) in SHM is now adding more advantages for monitoring displacement components (static, semi-static, and dynamics) of structures with high sampling frequency [2,4,5]. GPS serves one of the best sensors for deformation monitoring, because of its higher sensitivity and lower labor consumption than that of traditional geodetic survey techniques and other sensors [6,7]. But still limited use of GPS in the oscillations performance studies is observed, therefore, other sensors are attached to GPS monitoring system to detect the oscillations performance of structures. Moschas and Stiros [1] and Gorski [3] introduced and used the multi filter method to extract the dynamic performance of structures for the GPS monitoring system. In the present paper, a comparison between novel application techniques to reconstruct the waveform of GPS oscillations of structures to decrease the monitoring system cost is presented.

The advantages of GPS to monitor the deformation of civil structures are summarized in [2,3,4,8]. Nevertheless, the drawbacks and errors still affect the accuracy of the GPS measurements for the monitoring deformations. The contents of the dynamic displacement of the structures are almost masked by noise, while there are fundamental yet main spectral components. Moschas and Stiros [2] concluded that the noise characteristics of short duration records describing dynamic effects are not well understood may be because of the high rate of GPS sensors. The noises of short and long duration records for the high rate (≥1 Hz) of real-time GPS measurements include white, flicker, random walk, time-correlated, and colored noise [1,2,3,4]. Additionally, the error in GPS instrument sources like Multipath errors and self-noise of instruments used, are included [2,8]. This is the main reason for the difference in the GPS measurements’ outputs; also other digital instruments lead to significant differences in their output under the similar condition. Therefore, the noise characteristics should be studied to know its properties and characteristics.

Moschas and Stiros [1,2] represented the identification of signal that can be defined as a real challenge for the geodesy, structure and earthquake engineering. Linear and non-linear identification methods can be used to predict the displacement components of different structure applications [9,10,11]. Linear theory methods are widely used to study the structural behavior under small displacements around a safe state, but this approach does not provide information concerning other characteristics of non-linear systems, such as the magnitude and frequency of any limit-cycle oscillations. For this purpose, non-linear movement analysis is required. Nowadays, the neural networks (NN) can be designed and trained to perform complex functions and solve non-linear problems that are difficult for conventional computers and traditional solution methods [12]. The NN models seem to be highly precise prediction models [9]. NN consist of an input layer, a hidden layer(s) and an output layer. Each layer is formed by a number of nodes, and each node represents a neuron. Wang *et al*. [12] and Zhang *et al*. [13] summarized the design of NN as follow: NN models are composed of simple elements operating in parallel; a NN model can be trained to perform a particular function by adjusting the values of the weights between elements so that a particular input leads to optimal output [12]. The adjustment of NN depends on the comparison between the output and the target. NN aided adaptive and Kalman filters were used to smooth and filter the dynamic GPS monitoring data [14,15].

Jwo and Huang [14] applied a multilayered neural network to identify the measurement noise; numerical simulations show that based on the proposed approach the adaptation performance is substantially enhanced and the positioning accuracy is significantly improved. Kaloop and Kim [15] used the adaptive filter with NN to reduce the errors in the GPS monitoring system, and observed that the designed model can be used to remove the GPS noises for the long period of the movement components. Akyilmaz *et al*. [16] have used the NN to model the complex behaviors of bridge deformations based on multi-input multi-output model, and found that the used model has increased the accuracy of GPS observations. Kaloop and Hu [17] proposed a NN model for the damage detection of long span bridges based on predicting NN model for acceleration observations, and concluded that the NN model can credibly improve the prediction signals. Pantazis and Alevizakou [18] used NN to predict the vertical deformation of structures, and emphasized on the arguable possibility that the capabilities of NN can be successfully deployed to predict the dynamic behavior of modern and monumental structures. Chen *et al*. [19] concluded that the Back-Propagation Neural Network (BPN) ensemble filter cannot only reduce random additive and multiplicative white noise inside signals, but also preserve their characteristics. Moreover, they found that the BPN can be used to reduce the noises and improve the frequency contents of signals. El-Rabbany and El-Diasty [20] used NN models for de-noising Micro-Electro-Mechanical Systems (MEMS)-based inertial data, and inferred that the NN improved the accuracy of monitoring system based on de-noised original signals. Moreover, many studies have used NN models to predict a position of monitoring application with integrated GPS/inertial navigation system (INS) [21,22]. These previous studies have revealed that the strength of NN models can be used to predict and de-noise solutions of different monitoring sensor error problems. 

The present study has compared different NN models with and without aiding filters to optimize NN models applicable to de-noise the GPS dynamic measurements noises and to extract the displacement components of structures. From the previous studies, it can be seen that the NN models are used to predict and improve the position tracking of monitoring points in static and semi-static GPS-movement components. In this study, a novel application of NN model is investigated to improve the accuracy of the dynamic performance of structures. This paper is organized as follows; Section 2 presents the traditional NN models and with filter applications as well, Section 3 shows the models’ results and discussions based on simulation study and application for the better model detection with real-time GPS monitoring system, furthermore the comparison with previous method application is presented, and Section 4 encompasses the conclusion of the presented work.

## 2. Identification Models

**Figure 1:**
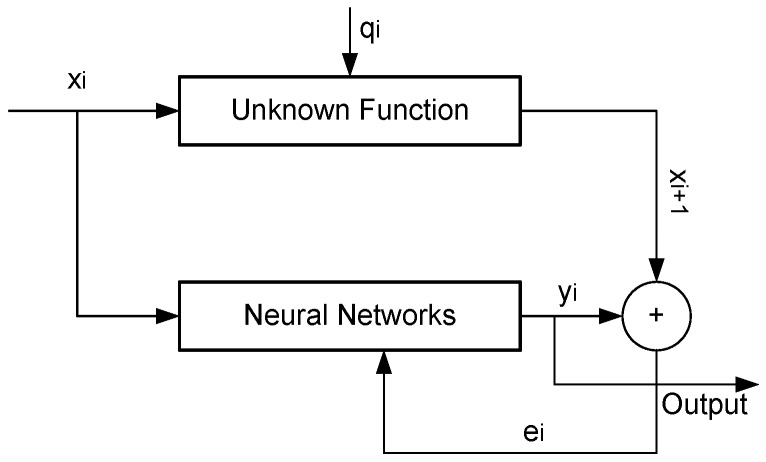
Design nural networks (NN) stochastic model identification system.

The stochastic NN models output only identification systems are derived from the state-space models, which expresses the equation of movement as a first-order differential equation, as shown in Figure 1. This equation can be discredited as in Equation (2), where the system is observed by Equation (1).

(1) x_(i+1)=Ax_i+q_i

(2) y_i=Cx_i+e_i

where x_i is state vector of structure’s movement responses from NN models; y_i is output vector at m locations on the structure and q_i and e_i are Gaussian-distributed process noise and measurement errors vector sequences matching the state and output responses. The coefficients A and C are known as state and output matrices, which are calculated based on NN method. The following models are used widely to predict, smooth and adjust time series of monitoring point’s positions with different applications [14,15,20,23,24].

### 2.1. Back-Propagation Neural Networks (BPN)

**Figure 2:**
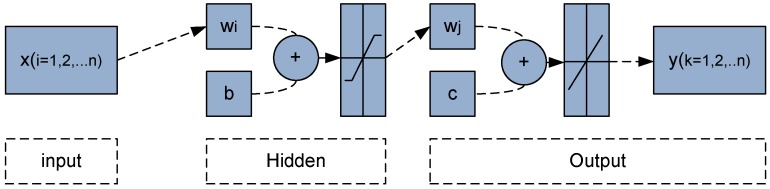
Back-propagation neural networks architecture.

This model is a type of feed forward artificial neural networks. In feed forward neural networks, the data flow is processed in one direction and the prediction output is obtained based on the current input sets. The architecture of BPN is shown in Figure 2. The method is selected based on the results included in Chen *et al*. [19] and presented earlier in introduction. The training algorithm depends on the weights assumed, initialized as small random values. In feed-forward stage the input signals (*x_i_*, i = 1,2,…n) assume an input unit and transmits these signals to the hidden units. Each hidden unit (*Z_j_*, j = 1,2,…m) calculates the sum of the weighted input signals, applies an activation function (*f*) and sends their result to the output unit [24]. The process of input signals to the hidden layer and output hidden layers can be represented as follow:

(3) $$Z_{j}=f(\sum_{i=1}^{n}{x_{i}w}_{i}+b)$$

In Equation (3) the term *b* and *w*_i_ are the bias on the hidden node and weight factor from input to hidden units.

The same for the output layer, the output of BPN can be represented as follow:

(4) $$y_{k}=f(\sum_{j=1}^{m}Z_{j}w_{j}+c)$$

In Equation (4) the term *c* and *w*_j_ are the bias on the output node and weight factor from hidden to the output units.

The weights and bias of the output node are updated using gradient-descent based delta-learning rule as follow [11,21]:

(5) $$\begin{array}{l} w_{j}(n+1)=w_{j}(n)+\Delta w_{j}\\ c(n+1)=c(n)+\Delta c \end{array}$$

Similarily, the weights and bias of the hidden nodes are updated as:

(6) $$\begin{array}{l} w_{i}(n+1)=w_{i}(n)+\Delta w_{i}\\ b(n+1)=b(n)+\Delta b \end{array}$$

where the weight and bias correction terms can be solved as:

(7) $$\begin{array}{l} \Delta w_{i}=\beta \delta_{i}(n)Z_{i}(n)\\ \Delta w_{j}=\beta \delta_{k}(n)y_{j}(n)\\ \Delta c=\beta \delta_{k}(n)\\ \Delta b=\beta \delta_{i}(n) \end{array}$$

where, *β* is the learning rate and *δ_k_*(*n*) and *δ_i_*(*n*) are the error coefficients at the output and hidden nodes, respectively.

The output error can be calculated by comparing the predicted values with the target outputs. The smaller output error is a better fit for the model. In case the error does not achieve the required accuracy, the network weights should be re-adjusted along the opposite direction of the network to the required minimum network error [13,25].

### 2.2. Cascade- Forward Back-Propagation Neural Network (CFN)

**Figure 3:**
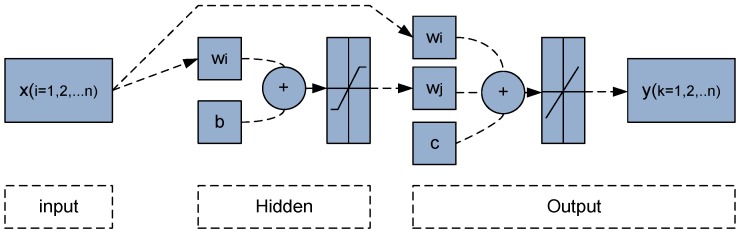
Cascade- forward Back-propagation neural networks architecture.

CFN model shown in Figure 3 is analogous to feed-forward networks, but it includes a weight connection from the input to hidden and output layers and, also, from each layer to the sequential layers. However, this process of the feed-forward networks with more layers and weights might learn complex relationships more quickly [26]. CFN model is similar to BPN in using the back propagation algorithm for weights generating and updating, but the main advantage of this network is that the output neurons are related to the input and hidden layer of neurons [21,26]. So, the output CFN is the sum of all previous layers as follows:

(8) $$y_{k}=f(\sum_{j=1}^{m}Z_{j}w_{j}+\sum_{i=1}^{n}x_{i}w_{i}+c)$$

### 2.3. Adaptive Filter Neural Network (ADFN)

**Figure 4:**
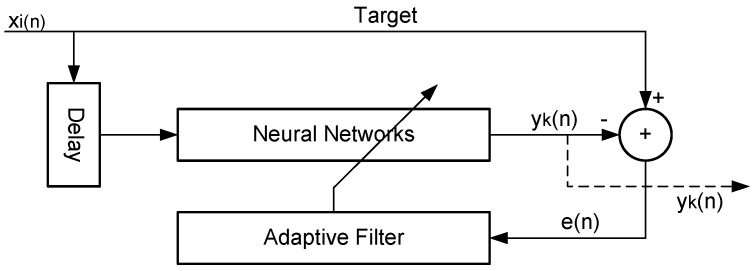
Adaptive filter neural networks architecture.

The adaptive filter aided neural networks can be used to reduce the sensor noises from unknown noise resources [15,27]. The operation of such an adaptive filter involves two basic processes: filtering process producing an output in response to an input sequence and an adaptive process for the control of adjustable parameters used in the filtering process [27,28]. In this model, feed forward artificial neural networks are used with adaptive filter to update the weights coefficients. Kaloop and Kim [15] used this model to de-noise the long period of bridge movement components. However, in this study, it is used to estimate the accurate short period displacement component. Figure 4 shows the ADFN architecture and the methodology, based on Equations (9) and (10).

(9) $$y_{k}=f(\sum_{i=1}^{M-1}w_{i}x_{n-i}+c)$$

(10) $$w_{i}(n+1)=w_{i}(n)+2\delta e(n)x(n-i)$$

where, *w* is the filter weight coefficient, δ is the step, *M* is the filter order.

### 2.4. Extended Kalman Filter Neural Network (EKFN)

The Kalman filter is a finesses tool to change the actual system parameters. Moreover, it has a better tracking ability to mutation status and lower sensitive to system noise, measurement noise and the initial statistical properties [22]. Nowadays, the extended Kalman Filter (EKF) has been broadly used in the application of nonlinear model parameters estimation for the neural networks [24]. In the EKF, the nonlinear terms are approximated to first order linear terms using Taylor expansion. This model used feed forward artificial neural networks with extended Kalman filter to update the weight coefficients of artificial neural networks model. Based on stochastic model in Equations (1) and (2) and considering a nonlinear system described in [11,29], it can be assumed an input output mapping of the *k*-th unit (*k* = 1,2,…*v*) of the *l*-th (*l* = 1,2,…*m*) layer as given in Equation (11).

(11) $$y_{k}(l)=f(\sum_{p=1}^{v-1}w_{p}(l)x_{p}(l-1)+w_{0}(l))$$

where *w_p_* and *w_0_* are coefficients and biases, these are lumped together into the weight vector *w*.

Now, let *ζ_t_* be the error made in approximating f by the NN, implementing the mapping λ for the input signal *x_t_*. Such a network can be represented as:

(12) $$\begin{array}{c} w_{t+1}=w_{t}\\ y_{t}=\text{λ}\left({\text{w}}_{\text{t}},x_{\text{t}}\right)+{\text{ζ}}_{\text{t}} \end{array}\text{t }=\text{ 1},\text{2},\ldots .\text{n}$$

Herein, the weights optimization used EKF to estimate the *w_t_* of the NN at times t = 1, 2,…n are as follow:

(13) $$w_{t}=w_{t-1}+K_{t}(y_{t}-\text{λ}\left({\text{w}}_{\text{t}-1},{\text{u}}_{\text{t}}\right))$$

where

(14) $$K_{t}=P_{t}C_{t}^{T}{\left(C_{t}P_{t}C_{t}^{T}+R_{t}\right)}^{-1}$$

(15) $${\text{P}}_{\text{t}+1}={\text{P}}_{\text{t}}-{\text{K}}_{\text{t}}{\text{C}}_{\text{t}}^{\text{T}}{\text{P}}_{\text{t}}^{\text{T}}+{\text{Q}}_{\text{t}}$$

*K_t_* is the Kalman gain matrix, *C_t_* is a matrix of derivatives of *λ_i_, R_t_* and *Q_t_* are the covariance matrix for the process noise and measurement errors as shown in Equations (1) and (2). Herein, in this model assumed *P_0_* and *R_t_* symmetric positive definite matrices, *Q_t_* a symmetric positive semi definite matrix, and Equation (13) is initialized with a given *w_0_* [29].

## 3. Results and Discussions

After the GPS data collections, the output of the GPS software is the time series of rover monitoring point coordinates in the World Geodetic System (WGS84). The coordinates of the monitored points are transformed into local coordinates with directions of monitoring members reflecting the apparent monitoring displacement. A long period displacement component of structures is a filter of apparent displacement which contains a semi-static displacement and background noise, whereas the short period displacement component, which supervises the long period component from apparent displacement, contains a dynamic displacement and some noise [2]. This study is focused on using the NN predictive models to de-noise the short period displacement components because of their significance in time and frequency modes’ calculations for the structural dynamics. A MATLAB simulation for noise reduction is used to simulate complex noise and verify the designed model.

### 3.1. Simulation Noise Results

**Figure 5:**
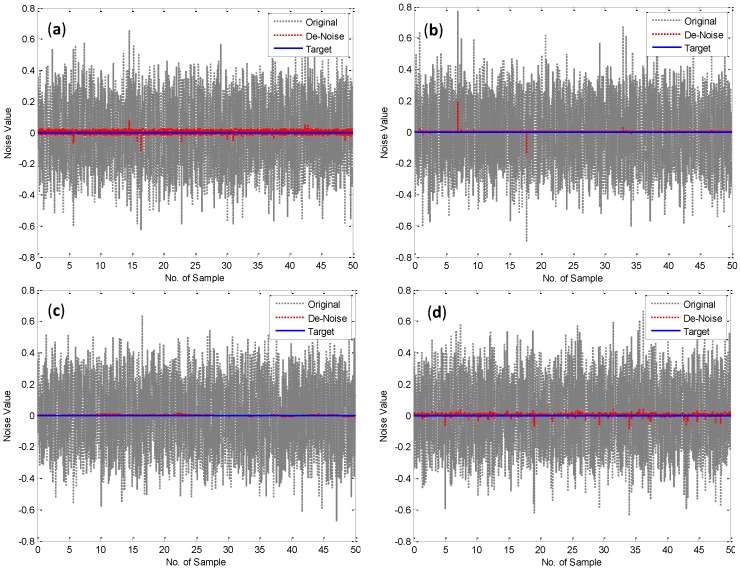
Comparison of the simulation results (**a**) Back-Propagation Neural Network (BPN); (**b**) Cascade- Forward Back-Propagation Neural Network (CFN); (**c**) Adaptive Filter Neural Network (ADFN); (d) Extended Kalman Filter Neural Network (EKFN).

Additive white Gaussian (AWG) noise is a basic noise model used in information theory to simulate the effect of many random processes that can occur naturally [10]. This noise can be modeled for satellite and deep space communication applications [10]. In this study, additive white Gaussian noise MATLAB code is used to create a noise as shown in Figure 5. The NN models designed in a previous section are tested to decrease the noise value created to zero as shown in Figure 5. The multilayer perceptron (MLP) network is considered with only one hidden layer hyperbolic tangent and one output linear activation function. The identification models are used to remove the noise affects based on predictive approach for the noisy signals. The signals models input are the AWG noise values and the predicted signal outputs were compared with target signal values. Three statistical evaluation criteria were used to assess and evaluate the models’ performances. First, the mean absolute error (MAE) is a linear scouring rule and describes only the average magnitude of the errors, ignoring their direction and second, the mean square errors (RMSE) describes the average magnitude of the errors by giving more weight on large errors. Third, the R-square is used to measure the fitness between target and predicted value, the R-square value closer to one indicates a better fit. Table 1 shows the MAE, MSE and R-square, which are used to test the accuracy of the model.

**Table 1:** Performance analysis for simulation de-noise using NN models.

Models	BPN	CFN	ADFN	EKFN
MAE	0.0093	8.65 × 10^−4^	0.0024	0.0037
MSE	1.22 × 10^−4^	1.206 × 10^−5^	7.98 × 10^−6^	3.174 × 10^−5^
R-Square	0.987	0.986	0.997	0.992

From Figure 5 and Table 1, it can be seen that the NN de-noise models increased the MSE accuracy of signals by 2.76%, 28.00%, 42.40%, and 10.60% for the BPN, CFN, ADFN and EKFN, respectively. In addition, it can be shown that the four models are affected by the noise peaks. Furthermore, the EKFN model is more affected by signals’ peaks as the EKF depends on the previous input signal as shown in Equation (12). The figure illustrates that the low and high matching values between target and de-noised signals occurred with BPN and ADFN models, respectively. From Figure 5 and Table 1, it can be noticed that the high R-square (0.997) and lower MSE (7.98 × 10^−6^) values are shown with ADFN model. Therefore, it can be considered that the ADFN is the best model to predict the true position of the sensors’ monitoring for the noisy signals. Meanwhile, it can be shown that the models CFN and EKFN performed as second best, due to high fitting shown and low absolute and mean square errors for the two models. In addition, the CFN gives a quite low value of MAE (8.65 × 10^−4^). Therefore, the CFN, ADFN and EKFN models have shown better results than BPN which is recommended by Chen *et al*. [19]. In addition, the results show clearly that the NN model filter can be efficiently used to remove the white noise signals for the random process of structures. It implies the multi-step filtering procedure can be constrained to de-noise the noisy sensor recordings for the structural oscillations and to determine its oscillation amplitude and modal frequency; this outcome complies with the results obtained by Moschas and Stiros [1]. Based on these results, it can be concluded that the ADFN served most effectively to predict the accurate monitoring for the noisy signals, therefore, the de-noised signals can be used to evaluate the movements of structures. The next section contains a case study to investigate this conclusion.

### 3.2. GPS-Bridge Movement Application: Case Study

**Figure 6:**
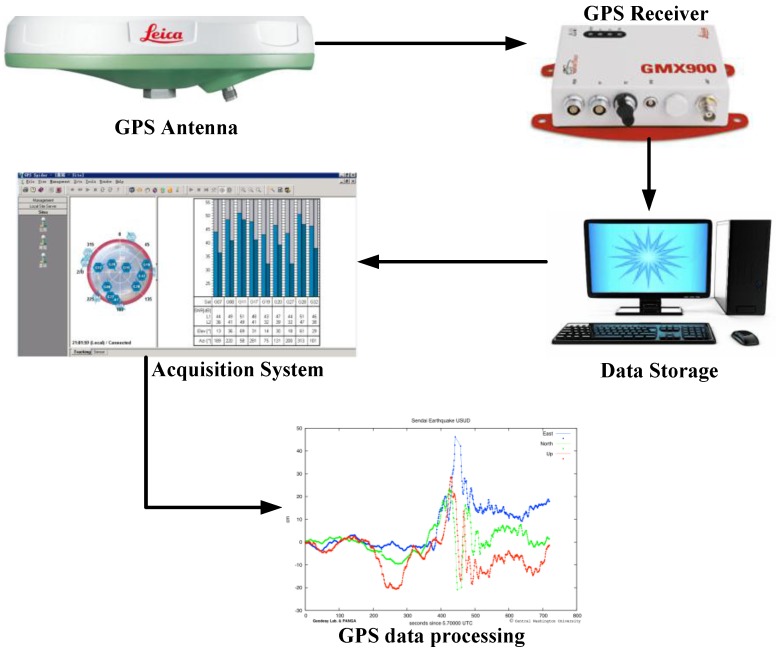
Global Positioning System (GPS) network diagram.

In this section, a GPS monitoring system of Zhujiang Huangpu [15,30] bridge located in China (Figure 6) is used to validate the ADFN model. This bridge is composed of a 705 m long cable-stayed span and a 1108 m long cable-suspended span. The monitoring system as shown in [30] contains 10 deck and three tower monitoring points with one base point as reference station to refresh the real time kinematic (RTK) correction message for the monitoring points. The sampling frequency of GPS used is 1.0 Hz to decrease the cost of the monitoring system. The observations were split into single hour observations for the processing purposes. The distance between base station and mid span point of cable-suspended bridge is 1.00 Km. The reference station refreshes the RTK correction messages for the GPS monitoring stations with a frequency of (1.0 Hz) via the optical fiber communication system. LEICA Geo-systems antennas and dual-frequency GPS receivers were used to observe the GPS satellite signals; the satellite elevation cut-off angle is 13°, for at least nine satellites. The data collected are mixed with that generated by the receivers own oscillators, and then LEICA Spider 2.1 RTK software was used to process RTK-GPS data. The data collected are processed coordinates in WGS-84 and converted to the local coordinate system by the coordinate transformation [15]. A local bridge coordinate system was established to be used in the analysis and the evaluation of the observed data. The x data represent the relative displacement changes along the longitudinal direction of the bridge; the y data represent the relative displacement changes along the transverse direction of the bridge while the z data represent the relative displacement changes along the vertical direction of the bridge. The relative displacement changes of the bridge deck are in millimeters. The outputs from GPS monitoring stations include monitoring point numbers and RTK GPS three dimensional coordinates, GPS time, satellite status data, GPS receiver status data, and so on, and the raw GPS outputs, as shown in Figure 6.

**Figure 7:**
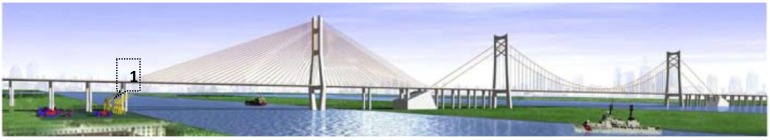
General view of the bridge and selection of the monitoring study point.

In this study, a fixation monitoring point is used (point 1), as shown in Figure 7, to study the effectiveness of the ADFN model on de-noising the GPS measurement errors and detect the dynamic behavior of the bridge monitoring point for the short period displacement component in both time and frequency domains. The selection point is not to provide any rotational restraints; the x-direction is flexible while any movement in z-direction is not permissible. Therefore, the GPS movements in three directions are small and the short-displacement component and vibration contents are effective.

**Figure 8:**
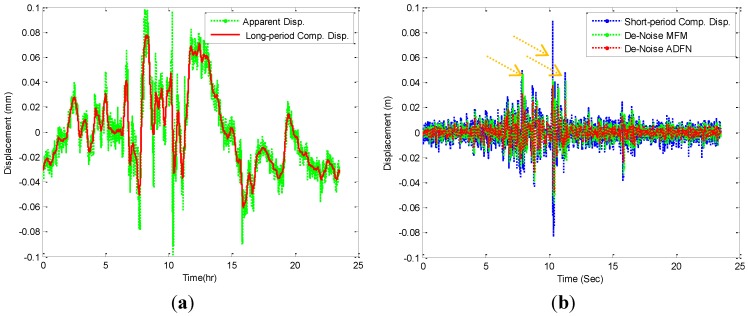

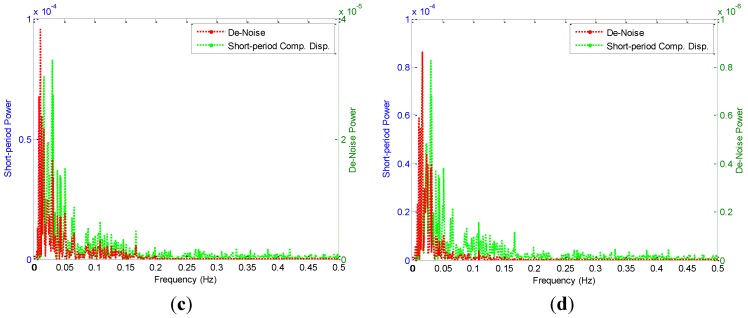
Dynamic performance for z direction. (**a**) Long period displacement; (**b**) Short period displacement; (**c**) Multi filter method (MFM) de-noise frequency contents; (**d**) ADFN de-noise frequency contents.

The moving average (MA) filter is used to extract the long period component of bridge performance. The MA filter is a simple low pass FIR (Finite-duration Impulse Response) filter, commonly used for smoothing an array of sampled data (Matlab, Release 12, Mathworks, Inc., Natick, MA, USA). In the usual case, the MA filter coefficients are symmetrical, and then such filters have linear phases, so they delay signals of all frequencies equally (Matlab, Release 12, Mathworks, Inc., Natick, MA, USA), since the long period of the movement components is important to identify. Herein, first the comparison between the introduced method for the dynamic performance of bridge by Moschas and Stiros [1], *i.e.*, multi filter method (MFM) and ADFN model, is made. Moschas and Stiros [1] used MA filter and an eighth-order Type 1 Chebyshev band-pass filter to extract the oscillation GPS high rate of rigid bridge performance. The z-direction for bridge selection point is more rigid than other direction movements. Therefore, the z direction is selected to make the comparison between the two models. Figure 8 illustrates the comparison between two methods based on the extracted long period of apparent GPS displacement of z direction, as shown in Figure 8a. The long period component is extracted by using a MA filter with span 855. Then, the short period displacement component is computed based on supervised long period component from apparent displacement. The last step is de-noising the short period component for the MFM used eighth-order Type 1 Chebyshev band-pass filter based on the following parameters: pass-band frequency 0.02 Hz, stop-band frequency 0.4 Hz, pass-band ripple 0.1 dB and data sampling frequency at 1 Hz. These parameters were selected to be compatible with the GPS sampling rate of 1 Hz; while, the ADFN model used is based on the NN predictive approaches only, as shown in Figure 8b. Therefore, the ADFN model simply uses and includes all oscillation monitoring frequencies of bridge. The frequency and power are calculated using Fast Fourier Transformation (FFT). The high pass filter is used to calculate the frequency for the monitoring point greater than 0.02 Hz, as shown in Figure 8c,d. Table 2 presents four statistical parameters that are MAE, MSE, standard deviation (STD) to measure the model’s accuracy and absolute maximum (Max) to measure the error’s effectiveness for the two models and original extracted signals.

Figure 8 and Table 2 illustrates that the statistical parameters for the ADFN model is smaller than MFM model. In addition, it is seen that the errors GPS reading generates, referred by arrow and shown in Figure 8b, are removed with ADFN model more precisely than MFM model. Moreover, the frequencies extracted from short-period components and two (MFM and ADFN) models noticed that the first mode frequencies are 0.017, 0.011 and 0.017 Hz, respectively. This means that the ADFM keeps the frequency model of original signal. In addition, it can be seen that the remaining noises and measurement errors are affected on the MFM between 0.05 to 0.15 Hz, while with ADFN model the noise frequencies are totally removed. Therefore, it can be concluded that the ADFN model is more effective and precise than MFM model for de-noising the short-period components of displacements in time and frequency domains. Furthermore, the de-noised signals by ADFN model can be used to analyze the dynamic performance of bridge.

**Table 2:** Statistical analysis of the short-period displacement component of z direction.

Parameter	Original Signal	MFM Model	ADFN Model
MAE (mm)	5.789 × 10^−3^	5.119 × 10^−3^	4.018 × 10^−3^
MSE (mm)	7.575 × 10^−5^	5.872 × 10^−5^	3.742 × 10^−5^
STD (mm)	8.703 × 10^−3^	7.663 × 10^−3^	6.117 × 10^−3^
Absolute Max (mm)	8.861 × 10^−2^	5.611 × 10^−2^	4.773 × 10^−2^

**Figure 9:**
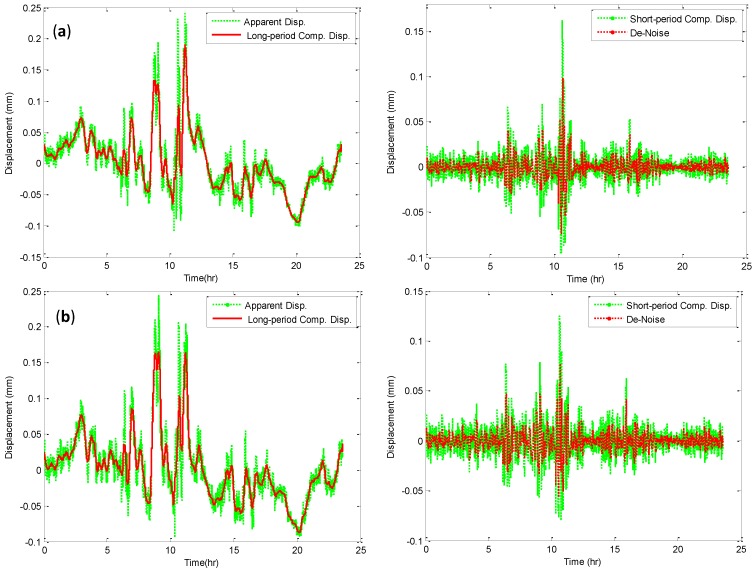
Long (left) and Short (right) period displacement for (**a**) x, (**b**) y directions.

However, the analysis of dynamic components of three directions of bridge GPS-movements can be evaluated based on extracting the de-noise signals and decreased the monitoring system cost. The following is the bridge evaluation based on GPS monitoring system. Figure 8a,b and Figure 9 show one day apparent displacement, long period and short period displacement components. Figure 8b and Figure 9, also, present the extracted long period component and de-noises signals for the apparent and short period component displacements by ADFN model.

From the fixation of the monitored point, and Figure 8a,b and Figure 9, it is seen that the displacement components in x and y directions are greater than z direction displacement. In addition, the standard deviation of x, y and z directions are 0.154, 0.174 and 0.033 mm for the apparent displacement, respectively. This means that the noises cover the apparent displacement is vibration and background noises. Also, the correlation between the apparent and long period components is high in the three directions of the monitored point. Therefore, the correlation between the three direction movements is high. It means that the effectiveness of the moving average is high in extracting the long period monitoring movements, and the state of structure is healthy. Also, the time history of short period displacement components for the x and y are greater than in the z direction, and the maximum short period for the x direction is 0.09 mm. This is primarily because of the monitored point fixation. From the short period displacement components and de-noising calculations, it can be seen that the model increased the accuracy of the short period displacements by 83.3% in the x and y directions and 93.8% in the z direction. From the time history of the long period component, it can be seen that the displacement of the monitored point is very small with a maximum displacement of 0.19 mm in the x direction. It is because of the available movement of this point in this direction. From these results, it can be concluded that the ADFN model can be permitted to de-noise the noisy GPS recordings of the oscillations of a stiff bridge points, and determine its oscillation amplitude and modal frequency.

**Figure 10:**
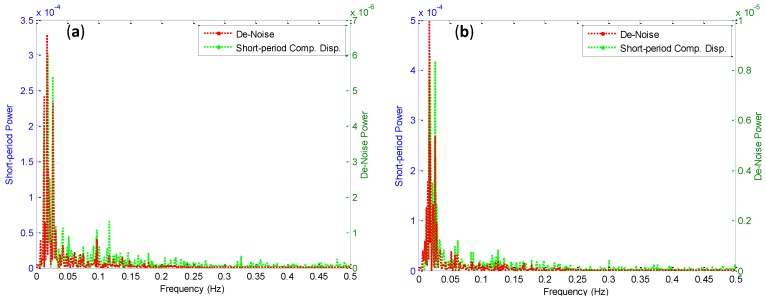
Frequencies of short period before and after de-noise GPS monitoring point (**a**) x, (**b**) y.

Figure 8d and Figure 10 illustrate the frequency calculation for the short period component and de-noises displacement. The power spectrum density of a signal is decreased to about 98% in the three directions for the monitoring point. This means that the high noise affected the low and high frequency modes calculation, and the noise can be misleading the frequency modes in high and low frequency modes. Also, the low frequency modes of short period displacement components values appeared in between 0–0.2 Hz. However, it is concluded that the spectral analyses revealed low-frequency colored noise, statistically significant below 0.2 Hz, and this result complies with the results obtained by Moschas and Stiros [2]. Also, the frequency models of short period components are clearer with de-noise signal, especially in the z direction. It means that this model is more adequate for frequency modes calculation.

Finally, this study reveals the potential of using GPS to measure the displacement history and the model frequencies of stiff monitoring point of structures. Model frequency up to 0.5 Hz is not included in this paper because of the limited availability of GPS monitoring sampling frequency. Also, the GPS measurements with a sampling rate of 1.0 Hz may underestimate the amplitude of displacement components. These problems, however, can be overcome with the use of modern high-frequency GPS. In addition, study for the availability of the NN models is used to de-noise the GPS vibration measurements in low frequency mode effects. These results are expected to have important implications in the structural health monitoring and the design of structures, especially for the low frequency modes analyze.

## 4. Conclusions

Neural Networks are used in this study to select the best model for improved operational health monitoring of structures based on GPS geodetic survey techniques. Four models are used to de-noise the GPS monitoring applications, which are Back-propagation, Cascade-forward back-propagation, Adaptive filter (ADFN), and Extended Kalman filter. An updated weight for NN models is used as the main factor to de-noise monitoring GPS applications. The results of the four models are compared to suggest and recommend the optimum de-noising model which can be used in GPS monitoring for measuring the deformation of structures. Furthermore, the recommended model is compared with previously used method (multi filter method (MFM)) to improve the model selection results with real monitoring of GPS data. The following conclusions are drawn from this study:

(1) The multi-step filtering procedure can be constrained to de-noise the noisy measurements of the oscillations of structures, and to determine its oscillation amplitude and modal frequency. In addition, it is concluded that the ADFN is the best model and thus suggested for use to de-noise the GPS measurements. In addition, the ADFN model is observed to be more effective and accurate than MFM model for de-noising short-period components of displacements of GPS real monitoring data in time and frequency domains.
(2) The apparent displacement measurements contain noises that can be considered as vibration and background noises. Moreover, the ADFN model increased the accuracy of short-period displacement by 83.3% in the x and y directions and by 93.8% in the z direction.
(3) The de-noised short-period displacement component of the measurements has decreased the power spectrum density to 98%. This means that the noise has high effect on the high and low frequency vibration modes of structures. From the frequency modes calculations, it is assumed that the low frequency modes of the short-period displacement component values are between 0–0.2 Hz.
(4) The GPS measurements with a sampling rate of 1.0 Hz may in fact underestimate the amplitude of displacement components, this problem, however, can be expected to overcome with the modern high-frequency GPS.
(5) The de-noised short-period displacement component based on NN predictive models is expected to have significant implications in the SHM and the design of structures in the low-frequency content.
